# Supplementary material for: The causal role of circulating inflammatory markers in osteoporosis: a bidirectional Mendelian randomized study
Source: Front Immunol. 2024 Jul 18;15:1412298. doi: 10.3389/fimmu.2024.1412298 (PMC11291241; doi:10.3389/fimmu.2024.1412298)
Supplement: Supplementary file 1 [file DataSheet_1.zip › supplementary material/CIM-OSTEOPOROSIS /GCST90274837.csv_forest.pdf]

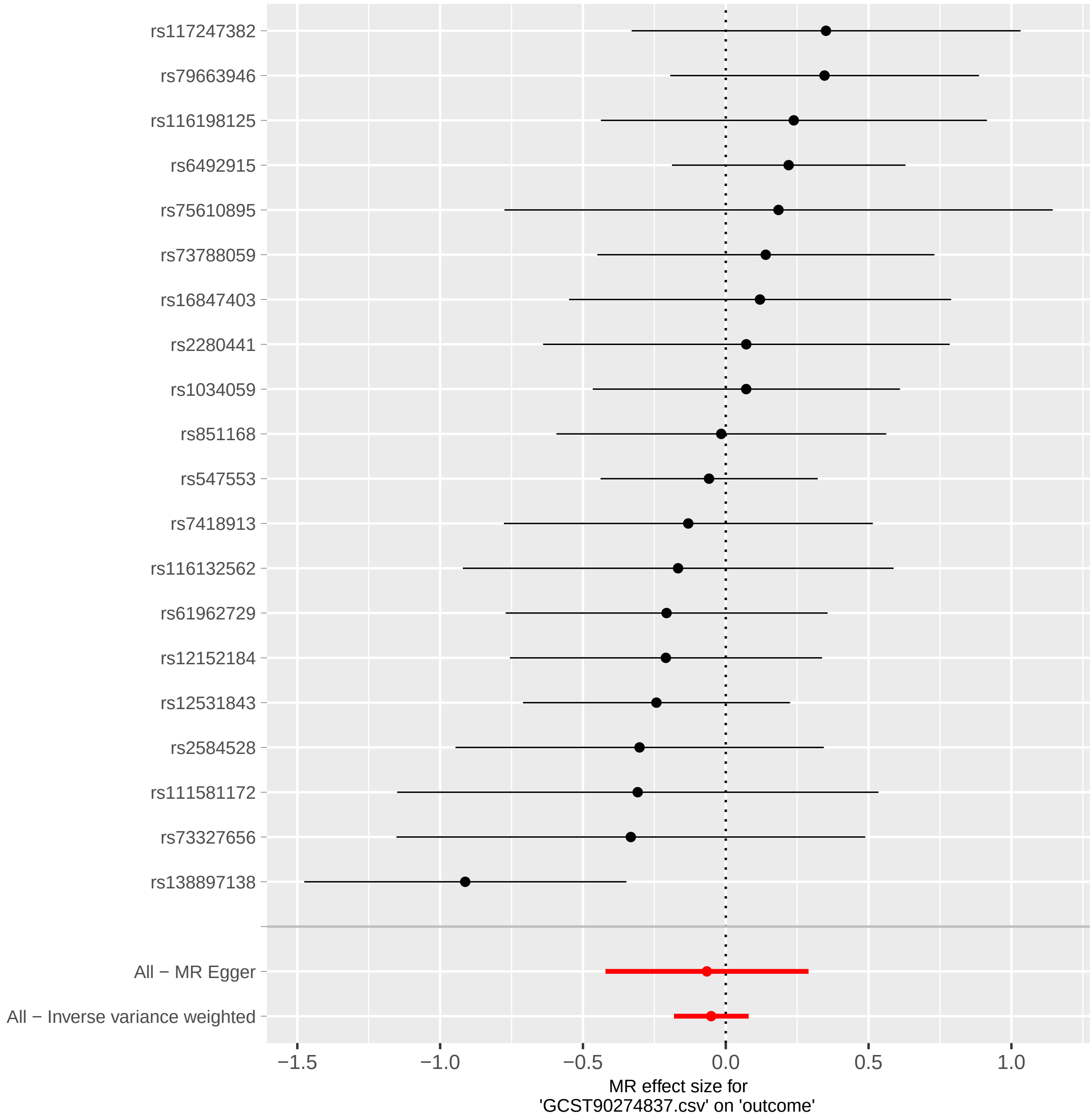
rs117247382
rs79663946
rs116198125
rs6492915
rs75610895
rs73788059
rs16847403
rs2280441
rs1034059
rs851168
rs547553
rs7418913
rs116132562
rs61962729
rs12152184
rs12531843
rs2584528
rs111581172
rs73327656
rs138897138
All − MR Egger
All − Inverse variance weighted
−1.5
−1.0
−0.5
0.0
0.5
1.0
MR effect size for
'GCST90274837.csv' on 'outcome'
